# The Effect of High-Intensity Ultraviolet Light to Elicit Microalgal Cell Lysis and Enhance Lipid Extraction

**DOI:** 10.3390/metabo8040065

**Published:** 2018-10-15

**Authors:** Thomas Sydney, Jo-Ann Marshall-Thompson, Rahul Vijay Kapoore, Seetharaman Vaidyanathan, Jagroop Pandhal, J. Patrick A. Fairclough

**Affiliations:** 1 Department of Chemistry, The University of Sheffield, Sheffield S3 7HF, UK; thomas_sydney@hotmail.co.uk; 2 Department of Chemical and Biological Engineering, ChELSI Institute, Advanced Biomanufacturing Centre, The University of Sheffield, Sheffield S1 3JD, UK; jo-ann.marshall@svgcc.vc (J.-A.M.-T.); R.V.Kapoore@swansea.ac.uk (R.V.K.); s.vaidyanathan@sheffield.ac.uk (S.V.); j.pandhal@sheffield.ac.uk (J.P.); 3 Department of Biosciences, College of Science, Swansea University, Swansea SA2 8PP, UK; 4 Department of Mechanical Engineering, The University of Sheffield, Sheffield S3 7HQ, UK

**Keywords:** microalgae, cell disruption, ultraviolet light, biodiesel, *Chlamydomonas reinhardtii*, *Dunaliella salina*, *Micractinium inermum*

## Abstract

Currently, the energy required to produce biofuel from algae is 1.38 times the energy available from the fuel. Current methods do not deliver scalable, commercially viable cell wall disruption, which creates a bottleneck on downstream processing. This is primarily due to the methods depositing energy within the water as opposed to within the algae. This study investigates ultraviolet B (UVB) as a disruption method for the green algae *Chlamydomonas reinhardtii*, *Dunaliella salina* and *Micractinium inermum* to enhance solvent lipid extraction. After 232 seconds of UVB exposure at 1.5 W/cm^2^, cultures of *C. reinhardtii* (culture density 0.7 mg/mL) showed 90% disruption, measured using cell counting, correlating to an energy consumption of 5.6 MJ/L algae. Small-scale laboratory tests on *C. reinhardtii* showed bead beating achieving 45.3 mg/L fatty acid methyl esters (FAME) and UV irradiation achieving 79.9 mg/L (lipids solvent extracted and converted to FAME for measurement). The alga *M. inermum* required a larger dosage of UVB due to its thicker cell wall, achieving a FAME yield of 226 mg/L, compared with 208 mg/L for bead beating. This indicates that UV disruption had a higher efficiency when used for solvent lipid extraction. This study serves as a proof of concept for UV irradiation as a method for algal cell disruption.

## 1. Introduction

Algal biofuels are the subject of large investment and a great deal of interest due to the promise of renewable, affordable, sustainable energy. Thus far, no company has achieved the full commercial potential that microalgae promise as a fuel source. Current processes to produce conventionally usable fuel from algae require numerous conversion steps. In particular, production of biodiesel from algae is severely limited due to the energy associated with cell disruption and lipid extraction. These processes can account for up to 26.2% [1] and 52% [2] of the energy input, respectively. The consequence is that the energy derived from the fuel is less than the external energy input required to process the algae [3]. Optimisation of algal cell disruption is thus an important step in the processing of algae for biochemical extraction, especially in biofuel applications [4]. Whilst various methods achieve cell disruption, they scale poorly on an industrial level. A low cost, scalable method for disruption is still sought. Such a technique would need to be low-energy, low-cost, continuously operable and, importantly, maintain the quality of the desired compounds extracted.

Conventional disruption techniques used to lyse algal cells ready for processing, such as homogenisation, microwave, sonication and bead milling, have a high associated energy cost and remain relatively expensive [5,6]. Techniques that disrupt algal cells must rupture the rigid cell wall in order to extract the commercially interesting compounds such as lipids and proteins. Additionally, these traditional techniques do not have the same disruption efficiencies on all species. Algae such as *Chlorella vulgaris* possess a thick cell wall which is highly resistive to mechanical stress, and as such, bead beating is less effective [7].

Lee et al. [8] compared five different methods, (including autoclaving, bead-beating, microwaves, sonication and a 10% NaCl solution) for cell disruption and concluded microwaves were the most effective method as this led to the largest lipid content extraction (44 mg/L). Unfortunately, the energy consumed amounted to 420 MJ per kilogram of dry algal mass which is a factor of over 4 times that of homogenisation [5]. This technique also proves difficult to optimise as the microwave energy is wasted on heating the extracellular water in the culture medium. Lee *et al*. [5] also compared bead milling, a simple technique for cell disruption. Beads are added to a culture, which is then vigorously shaken causing collisions between cells and beads; the beads erode the cell surface and cause lysis. This is slightly less effective than microwaves at cell disruption and higher in energy consumption at 504 MJ per kilogram of algal dry mass [5].

Many cell disruption methods deposit energy in the water rather than within the algae and are thus difficult to scale commercially. Bead beating is limited by losses due to viscous heating; microwave, sonication and other physico–thermal methods are limited by heating the water. Thus, this research explores the development of a low energy method to disrupt algal cells using ultraviolet (UV) light.

The short wavelength, high energy photons of ultraviolet B (UVB) and ultraviolet C (UVC) can lead to significant cell damage and are the most damaging wavelengths of UV light [9,10]. Ultraviolet A (UVA) is less effective, causing indirect damage to cells through the production of reactive oxygen species that may damage DNA, proteins and lipids [10]. UVB and UVC cause direct DNA damage through absorption of photons by DNA bases, resulting in chemical quenching and the formation of pyrimidine dimers in the sequence [11].

Few if any studies, to our knowledge, have looked at the effect of high intensity UV radiation for cell lysis, especially for the extraction of chemicals. Moharikar et al. [12] did study the effectiveness of UVC to induce apoptotic or necrotic pathways, but not from a biotechnological viewpoint. They conclude that UVC causes apoptotic and necrotic pathways in *C. reinhardtii* following sufficient exposure to UVC. Furthermore, although high doses of UV light lead to cell lysis, its use as a method of cell disruption in algae for lipid extraction has not received significant interest.

### 1.1. Cellular Signalling after Ultraviolet Irradiation

UV light induced cell lysis is due to the absorption of UV radiation by DNA, RNA, protein and lipids which can lead to structural damage and signalling/metabolic disorder [9]. DNA replication may be defective following UV exposure if the correct repair does not take place and may lead to mistakes in transcription and translation which result in protein synthesis with incorrect sequencing and misfolds [11]. DNA damage can be repaired after initial exposure; photorepair will occur if the algae are placed back into natural light, through the enzyme cyclobutane pyrimidine dimer photolyase [11]. In the work presented here, algae were stored in the dark following irradiation in order to reduce photorepair and maximise disruption. At low UV doses this repair mechanism can prevent lysis [11]. Additionally, other DNA repair methods such as excision repair and recombination repair are possible but cannot be prevented by dark storage [9]. At industrial scale, a sufficiently high dosage would avoid any repair that cells could undergo as they would be irreparably damaged. For instance, at 300 s at 9 (UVA) and 1.5 (UVB) W/cm^2^ irradiation, cell counts before dark storage (data not presented) indicated approximately the same number of non-viable cells counted 24 h later.

**Figure 1:**
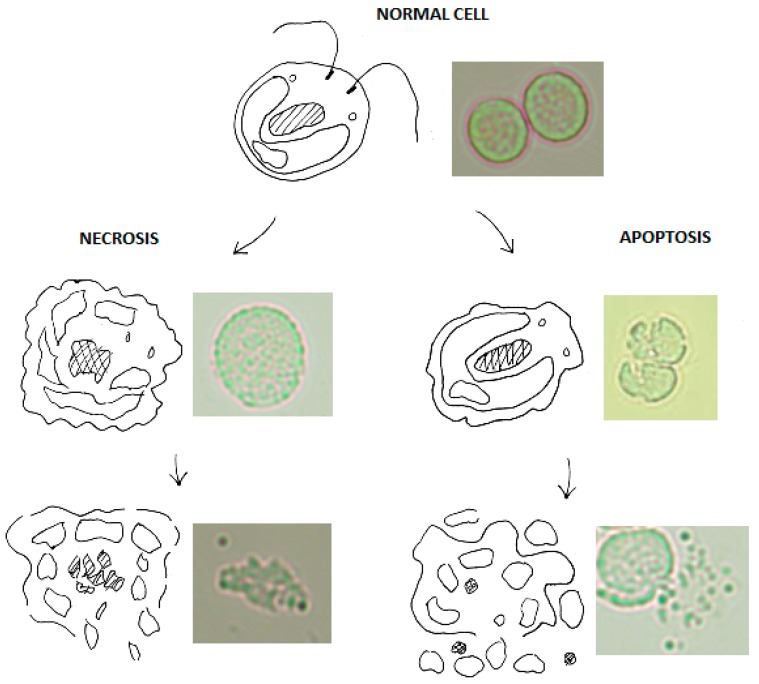
UV induced necrosis and apoptosis of *C. reinhardtii* cells visualised using Olympus BX50 microscope, 50× objective. Control image of no irradiation of a normal cell. UVB intensity at 1.5 W/cm^2^ for 300 s (equivalent to 450 J/cm^2^ UVB) for both necrosis and apoptosis. A 3 mL sample of *C. reinhardtii* in a quartz cuvette was exposed to 300 s of irradiation at 9 W/cm^2^ UVA (320–395 nm) and 1.5 W/cm^2^ UVB (280–320 nm) at a path length of 3 cm using a BlueWave 75 UV Curing Spot Lamp.

The cellular signalling cascade that leads to cell death following UVB exposure is complex and has not been fully investigated in algae. UVB radiation causes the formation of pyrimidine dimers in DNA which often lead to mutation of a cell’s genome. In mammalian cells, and shown here in algal cells, if sufficient mutations occur the cell may lyse through necrosis or apoptosis (see Figure 1); two pathways that operate differently but ultimately lead to cell death [13]. It has been previously shown that algae have a similar process [14]. Under intense trauma, the cell undergoes necrosis, the uncontrolled release of intracellular components. Cells which undergo this premature death rupture, and this is usually caused by mutations in genes which regulate key cellular processes. In contrast, apoptosis involves the regulated release of intracellular components and DNA fragmentation. It is often called programmed cell death due to its highly regulated nature and is activated if sufficient DNA mutations occur in particular genes. During apoptosis cells “pack” intracellular components into apoptotic bodies which are then released into the culture medium; some of which have high lipid content and can be referred to as lipid bodies.

In this work, the structural markers of apoptosis, necrosis and lipid bodies can be visualised using light microscopy and were present during cell counting experiments (see Figure 1). Mutations arising from UV radiation can elicit both necrotic and apoptotic pathways due to the random nature of base mutation. Importantly, the mechanism of cell death initiated by UV radiation should work consistently with any species of algae, as it attacks an organism’s DNA. There will be varying degrees of effectiveness due to different cellular characteristics and some species may have developed more complex defences to UV. However, given a sufficiently high UV dose these defences can be overcome.

### 1.2. Ultraviolet Light Compared to Conventional Disruption

Ultraviolet light as a method for algal cell disruption is a non-mechanical technique which differs from most conventional methods. There are other disruption techniques that utilise non-mechanical means such as chemicals, enzymes or microwaves but ultraviolet light is particularly effective because it targets DNA specifically, effectively shutting off an organism’s ability to function [13]. Additionally, water is highly transparent to UVB and hence no energy is wasted in treating the water; only the algae are affected [15].

This technology is readily scalable as UV sterilisation for water treatment is commonly employed on a commercial scale, with 8000 municipal systems currently operational; the largest of which is situated in the USA, sterilising 2.24 billion gallons per day [16].

Irradiating a non-dewatered culture of algae for disruption could be as simple as running a culture past a large UV source. This could be optimised by controlling the flow rate and maximising surface area through the use of mirrored surfaces and large surface area plates. Another example of industrial UV treatment of water is the bottled water industry which requires as little as 10 kWh per million litres [17].

The use of ultraviolet light as a method of cell disruption on algal cells is explored here. Three species with contrasting cell wall characteristics (*C. reinhardtii*, *D. salina* and *M. inermum*) were irradiated with UV light at various durations to determine cell disruption efficiency via light microscopy. *D. salina* lacks a cell wall, *C. reinhardtii* has a reasonably durable multi-layered glycoprotein-based cell wall and *M. inermum* has a thick cell wall [18,19]. Lipid extraction using solvents and transesterification on irradiated samples was also undertaken as a suitable measurement method to translate product yields to cell disruption.

## 2. Results and Discussion

### 2.1. C. reinhardtii Irradiation

Following irradiation, morphological changes to *C. reinhardtii* cells were apparent and showed that the majority of cells were no longer viable at the longer exposure durations. In particular, loss of a well-defined cell wall at the boundary between the cell and extracellular media indicated cells were not viable and damaged beyond repair. Often cells appeared as clusters of beads which is most likely an indication of the formation of apoptotic bodies (Figure 1). Trypan blue was used as a stain, thereby identifying cells with compromised walls, though sometimes cells were not stained but were no longer viable due to fragmentation.

Viable and non-viable cell counts were not possible with the trypan blue stain as it did not always bind to cells. Thus, an effort was made to only count cells as non-viable if it was unmistakable as not to overestimate the efficacy of UV disruption. Additionally, most intact cells were swollen, indicating a loss of osmotic control leading to an influx of surrounding media, perhaps due to cell wall damage or even a critical mutation in the cells homeostatic regulatory genes. 

Highly bleached cells indicative of a loss of chlorophyll were also present. Both swollen and bleached cells were likely non-viable due to imminent cell lysis through accumulation of damage beyond repair. However, it should be noted that in general, morphological changes were distinct enough to rule out any concern over bias reporting; Figure 1 shows unstained cells with distinct changes.

At higher exposure times of 150 s and more so at 300 s, a large amount of cell debris was present which indicated many cells had broken apart completely. This is likely due to cells undergoing necrosis or apoptosis, as can be seen in Figure 1.

**Figure 2:**
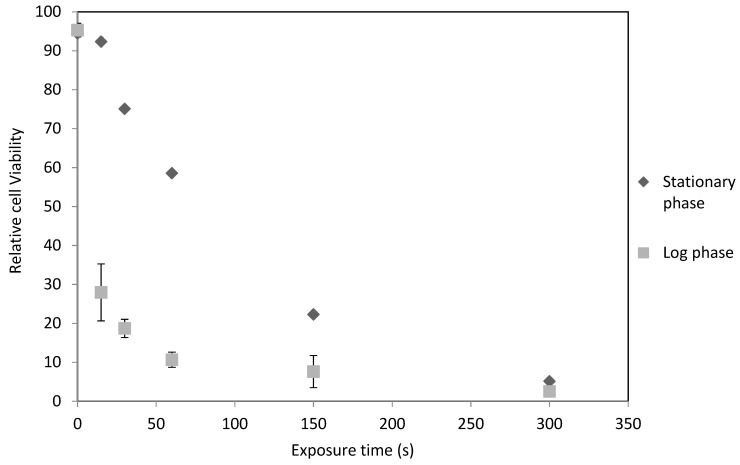
Relative cell viability of *C. reinhardtii* at various exposure times to ultraviolet light in the stationary and active growth phases. Stationary phase *n* = 1, log phase *n* = 3. Error bars represent the range for log phase. Control at 0 s of irradiation. UVB intensity at 1.5 W/cm^2^. Carried out as described in Materials and Methods. Culture densities approximately 0.7 mg/mL dry weight.

*C. reinhardtii* cells were compared at different growth phases to determine if there was a difference in UV cell disruption efficacy. Experiments with *C. reinhardtii* in stationary phase showed an exponential decrease in cell viability as exposure time increases, as shown in Figure 2. Lysis of 50% of the cells occurred at 71 s and 90% occurred after 232 s (equivalent to 348 J/cm^2^ UVB). 

A log phase *C. reinhardtii* culture under UV irradiation had a severe decline in cell viability with increasing exposure time. In this case, there was a prompt initial decline in cell viability as seen in Figure 2. Here 50% of algae cells were non-viable after 34 s and 90% after 127 s (equivalent to 190.5 J/cm^2^ UVB).

The more severe decline of log phase cells compared to stationary phase may be due to increased vulnerability of algal DNA during cellular fission, although this has not been investigated by the scientific community thus far.

### 2.2. D. salina Irradiation 

Viable and non-viable cells of *D. salina* were more distinguishable between one another than *C. reinhardtii* cells. Once again clusters of apoptotic bodies, swollen cells and a loss of a well-defined cell wall were visible, as seen in UV exposed *C. reinhardtii.* However, it was not possible to use trypan blue as a stain as it became apparent it had low miscibility with the saline media and unfortunately caused non-viable cells to aggregate. 

The lack of a suitable stain proved insignificant as the cellular markers for viability were evident. *D. salina* cells are typically bright green and resemble a tear drop. They also are highly motile, and possess large flagella. These marked characteristics are easy to recognize and any changes due to UV radiation were distinct. After exposure, cells were more spherical and at high exposures there was a discernible reduction in motility, often rendering them non-motile. Increased cell debris was present at high exposure times as in the case of *C. reinhardtii*, indicating many cells have been completely destroyed and thus do not show up in cell counts. 

**Figure 3:**
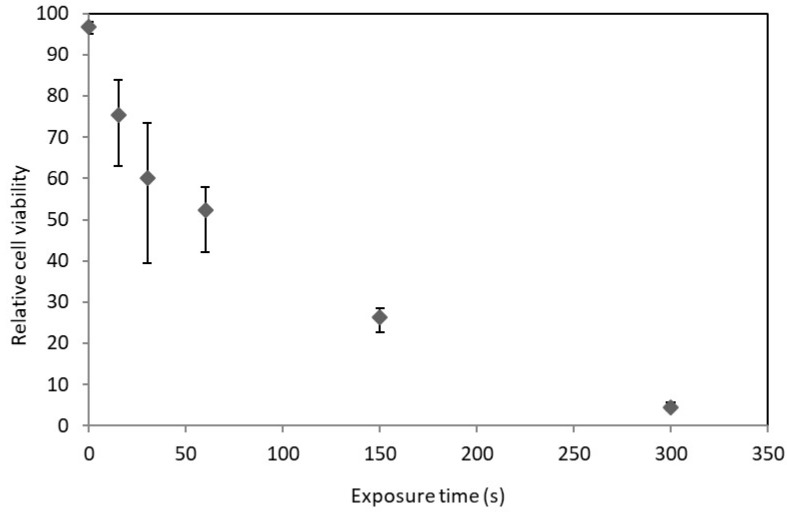
Relative cell viability of *D. salina* at various exposure times to ultraviolet light in the stationary phase. *n* = 3. Error bars represent the range. Control at 0 s of irradiation. UVB intensity at 1.5 W/cm^2^. Carried out as in Materials and Methods. Culture densities approximately 0.7 mg/mL dry weight.

*D. salina* was irradiated in its stationary phase and Figure 3 shows the relationship between UV exposure and cell viability. Viability was similar to that of *C. reinhardtii*, with *D. salina* appearing marginally more susceptible to UV radiation.

It was anticipated that *D. salina* would be far more susceptible to UV radiation than *C. reinhardtii* due to the difference in cell wall chemistry. However, due to the mechanism of UV disruption, cell wall characteristics may not affect its disruption efficacy. UV light does not need to interfere with the cell wall to cause DNA damage (although it can damage cell wall lipids and proteins as well) which can cause death through necrosis or apoptosis. This indicates that even thick cell walled genera such as *Chlorella* could be candidates for algal biotechnology or biodiesel production using UV radiation as a disruption method [18].

### 2.3. UV Radiation as a Disruption Method for Biodiesel Production

While the UV light source used in this experiment contains UVA and UVB, UVA wavelengths do not cause direct DNA damage and hence have a much-reduced effect on cell disruption. As discussed in the Introduction, UVB and UVC can lead to significant cell damage and are the most damaging wavelengths of UV light. Therefore, this study highlights UVB light as the source for eliciting microalgal cell lysis.

To this end, samples of *C. reinhardtii* were irradiated with UVB radiation (1.5 W/cm^2^), alongside bead beaten samples as a control, before lipid extraction and transesterification to determine efficacy of UV radiation as a disruption method for biodiesel production. A detailed explanation of the experiment is within the methods section. Samples of *C. reinhardtii* were irradiated for 300 s or bead beaten. The samples were then solvent extracted with methanol:chloroform mixture (1:2) before heating at 80 °C for 90 min with 10% BF_3_/methanol. Following this the samples in hexane were submitted for GC-FID based FAME analysis using a TR-FAME capillary column.

**Figure 4:**
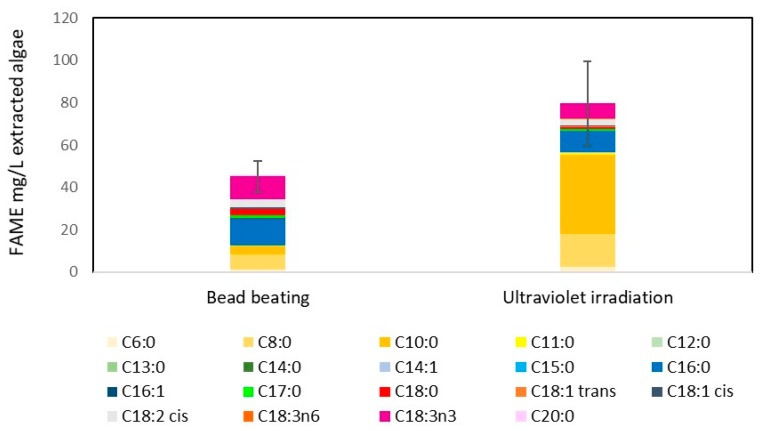
Comparison of biodiesel yield from disruption methods used on nitrate stressed *C. reinhardtii*. Fatty acid methyl ester yields from both bead beating and ultraviolet light irradiation disruption is shown. Yields represent transesterified lipids from 1 L of culture. UVB intensity at 1.5 W/cm^2^ for 300 s (equivalent to 450 J/cm^2^ UVB). Irradiation and bead beating carried out as in Materials and Methods. *n* = 2. Error bars represent the range. The legend indicates different FAME chain lengths.

FAMEs produced from *C. reinhardtii* samples that underwent lipid extraction and transesterification are shown in Figure 4, demonstrating that the new UVB radiation method was more effective than bead beating, bead beating having 45.3 mg/L culture and UV irradiation having 79.9 mg/L culture (approximately 0.7 mg/mL algal culture density). Meaning an approximate FAME yield of 7.7% and 11.3% for bead beating and UV irradiation, respectively. This indicates that ultraviolet light is an effective method to disrupt algal cells for biodiesel production. The simplicity of using light for disruption has great potential for scaling the technology to an industrial level. In fact, flowing a culture past an ultraviolet bulb could be a simple continuous way to disrupt a culture. 

### 2.4. Effect of Nitrate Stress on FAME Yield

**Figure 5:**
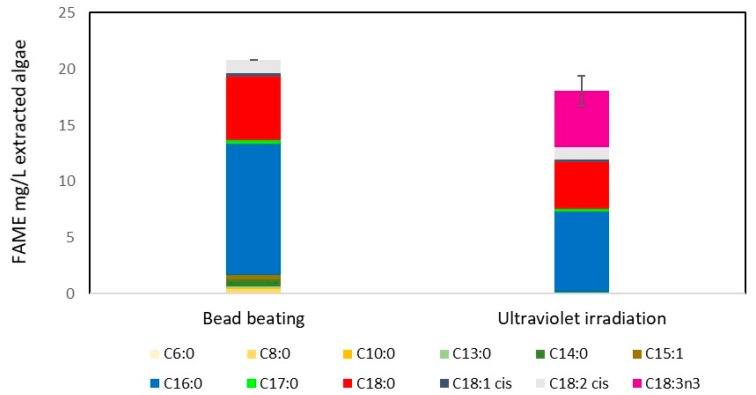
Comparison of biodiesel yield from disruption methods of nitrate rich *C. reinhardtii* culture. Fatty acid methyl ester yields from both bead beating and ultraviolet light irradiation disruption is shown. Yields represent transesterified lipids from 1 L of culture. UVB intensity at 1.5 W/cm^2^ for 300 s (equivalent to 450 J/cm^2^ UVB). Irradiation and bead beating carried out as in Materials and Methods. *n* = 1 for bead beading, *n* = 2 for UV irradiated samples. Error bars represent the range. The legend indicates different FAME chain lengths.

**Figure 6:**
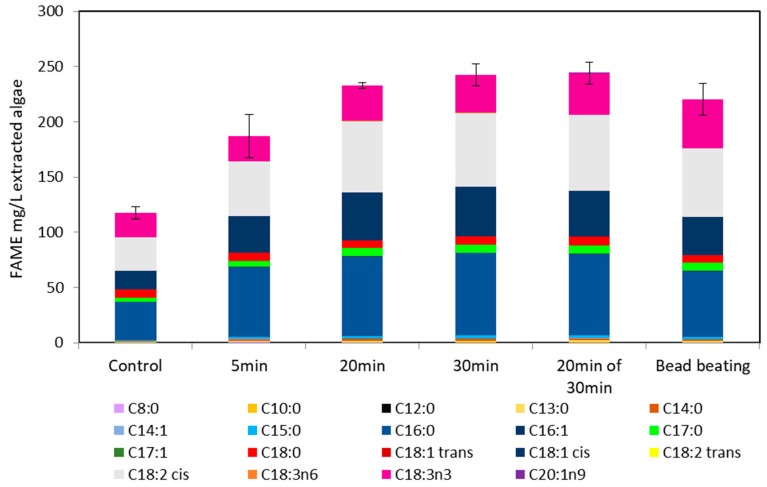
Comparison of biodiesel yield from UV light exposed *M. inermum.* Fatty acid methyl ester yields from both bead beating and ultraviolet light irradiation disruption is shown above. Yields are scaled to represent transesterified lipids from 1 L of culture. UVB intensity at 1.5 W/cm^2^. 20 min over 30 min represents 40 s of irradiation per min for 30 min. Irradiation, bead beating, and control carried out as in Materials and Methods. Error bars represent the range. *n* = 2. The legend indicates different FAME chain lengths.

Comparing UV irradiated samples and bead beaten samples from a culture containing nitrate (Figure 5) with a nitrate stressed culture (a common method used to increase lipid yield) (Figure 4), FAME yield was considerably higher in the nitrate stressed system (Figure 4), UV irradiated samples having higher yields. The major difference between the FAME yield was an increase in C8 and particularly C10 chain lengths. Interestingly this difference is not the same in the nitrate rich system (Figure 5), though as the FAME levels detected in were low, it is difficult to draw any firm conclusions other than that both UV irradiation and bead beating have a similar lipid extraction efficiency when intracellular lipid content is low. Typically FAME produced from algae is high in the C16 and C18 chains, which are the most suitable chain lengths for biodiesel [20]. The FAME profiles of UV irradiated algal cells generally follow this trend where the majority FAME peaks are C16 and C18 (as shown in Figure 6 and unreported data). 

### 2.5. Ultraviolet Light Irradiation of M. inermum

Following the irradiation of stationary phase *C. reinhardtii*, it became apparent that whilst it is a useful species for study due to its growth characteristics and lipid yields, its electrostatically linked glycoprotein cell wall is not as robust as other species of algae [18]. In particular, species such as *Chlorella* possess a thick covalently linked cell wall that often has to be freeze-thawed several times to lyse [18]. *M. inermum* is another species which is similar to *Chlorella* and has a thick cell wall [19]. Previous figures (1, 2 and 3) demonstrate that ultraviolet light is capable of disrupting both *C. reinhardtii* and *D. salina,* however, both lack thick cell walls that are difficult to disrupt. Therefore *M. inermum* was exposed to ultraviolet light at various dosages to determine its efficacy for cell disruption and for FAME yield following lipid extraction and transesterification.

Figure 6 shows that with increasing UV irradiance, FAME yield from *M. inermum* increases. The duration of exposure required to maximise lipid extraction is much longer than seen with *C. reinhardtii.* This is likely due to the increased cell wall thickness and covalent nature. Bead beating was performed for 2 min cycles fifteen times as opposed to 1 min, to ensure the cells were sufficiently disrupted. 

Furthermore, Figure 6 indicates the irradiation duration required was much higher but ultimately successful due to the mechanism by which UV light causes cell disruption, primarily through DNA damage. The smaller, thick cell walled *M. inermum* cells were successfully disrupted using UV light, given a high enough duration. The data presented in the cases of both *C. reinhardtii* and *M. inermum* suggests UV radiation to be a more effective cell disruption method than bead beating for FAME yield. Specifically, the FAME yields from *M. inermum* indicated UV radiation (30 min) achieved a FAME yield following transesterification of extracted lipid of 226 mg/L extracted algae compared to 208 mg/L extracted algae for bead beating.

### 2.6. Ultraviolet Light Cell Disruption Energy Efficiency

In order to determine the efficacy of a cellular disruption technique, the energy cost must be weighed against the disruption efficiency, especially for a biofuels application. The Bluewave DYMAX curer light source draws 75 W of power to function. Approximately 225 s of irradiation results in 90% cell lysis of a 3 mL aliquot of *C. reinhardtii*; this translates to an energy consumption of 5.6 MJ/L algae or 8 KJ/mg algae. It should be noted this is for a UV irradiation path length of only 3 cm with an approximate culture density of 0.7 mg/mL. Should a culture be concentrated through centrifugation, it is probable that higher efficiencies could be achieved using appropriate intensity, duration and path length. Eventually, a limit will be received where path length or culture density becomes too great for UV to pass through the culture, and this needs to be determined in future to allow scale up of the technique.

This is considerably lower energy (approximately 13 times) than other methods as shown by McMillan et al. [21], such as microwave treatment, which achieved 94.92 ± 1.38% lysis with an energy consumption of 74.6 MJ/L algae (1.8 × 10^8^ cells/mL). It should be noted that the above ~95% disruption efficiency was achieved with *Nannochloropsis oculata*, a smaller alga with a resistive cell wall. However, the fact that UV irradiation does not need to mediate the cell wall to cause disruption suggests disruption efficiencies will be similar across many species. Disruption efficiencies for *D. salina* and *C. reinhardtii* are comparable in their stationary phase and support that theory. This energy efficiency could further be improved upon through the use of reflective surfaces, more efficient bulbs or balancing irradiance intensity against duration.

### 2.7. Limitations of the Study

This study aims to present the use of UV light as a proof of concept method for cell disruption of algae to enhance lipid extraction. This novel method will need to scale in order to be industrially viable, however, this study was not aimed at exploring scalability. To this end, additional data is required in order to determine industrial relevance. Said data must detail the effect of UV light on cell disruption as a function of increasing cell density with costs and efficacy compared. A methodology designed with that in mind will further elucidate UV light’s industrial potential as an algal cell disruption method for biodiesel production.

Additionally, as this study is focused on biodiesel production, chain length and degree of saturation of fatty acids are an important measure of FAME quality. Due to the proof of concept nature of the study these aspects have not been directly addressed, however, they are important factors when determining scalability and industrial relevance of the novel method. While this study does not directly focus on FAME quality, is it of note that the FAMEs obtained using UV disruption of *M. inermum* do not have any notable difference in FAME quality (Figure 6). This is in contrast to *C. reinhardtii* FAMEs (Figure 4 and Figure 5), where there is a distinct increase in C8 and C10 esters, and in the degree of unsauration. Therefore, at this stage it is difficult to conclude whether UV disruption has an effect on FAME quality, but it is clear that additional studies need to explore the quality of biodiesel produced.

### 2.8. Future Work

Following this work various other studies will further investigate the use of UV light as an algal disruption method to enhance lipid extraction for biodiesel production. The initial experiments using UV light disruption were performed on *C. reinhardtii* as it is a model species; in general, it does not produce high lipid yields, however, for the purposes of determining if the technique was viable, it was a suitable candidate.

This proof of concept study was focussed on the viability of UV light as a disruption method rather than on maximising lipid yield using high cell density cultures (and therefore high lipid/FAME content). In order to investigate UV disruption for higher FAME yields, higher cell density cultures would be required which present different scalability challenges that are outside the scope of a proof of concept approach.

Future research should be aimed at determining the effectiveness of UV disruption for lipid extraction using high lipid producing algae with high cell densities. Such research would be more suitable in a study that is aimed at determining the scalability of the technique as well as optimising the irradiation duration, concentration and efficacy over a wider range of algae.

Additional work will also include the use of concentrated solar radiation as the ultraviolet light source for disruption of microalgal cells. Another study will explore the use of novel recyclable magnetic nanoparticles that can extract the disrupted algae and lipids following UV light disruption.

## 3. Materials and Methods

### 3.1. Algae Strains and Cultivation

*Chlamydomonas reinhardtii* (wild type CCAP 11/32A), *Dunaliella salina* (wild type CCAP 19/30) and *Micractinium inermum* were grown in 250 mL conical flasks with TAP medium, Vonshak’s medium [22] and Bold’s Basal Medium, respectively, at 25 °C under magnetic stirring and positioned approximately 10 cm from 36 W fluorescent tube light sources [22,23]. *Micractinium inermum* was isolated from an environmental sample at the University of Sheffield [24]. 

Cells were grown up to approximately 0.7 mg/mL dry weight. These species were selected as suitable organisms for UV irradiation studies as their broad background literature and contrasting cell wall characteristics would give insight into the effectiveness of UV radiation to lyse cells regardless of cell wall strength.

Cells were nitrate stressed, when required, by re-inoculating a late log phase culture in nitrogen free TAP medium, which was made by omitting ammonium chloride from the TAP medium recipe [25].

### 3.2. Ultraviolet Light Irradiation of Algae

Various 3 mL samples of *C. reinhardtii* (24 samples)*, D. salina* (15 samples) and *M. inermum* (8 samples) in a quartz cuvette were exposed to a series of irradiation periods ranging from 15 s to 300 s (5–30 min for *M. inermum)* with 9 W/cm^2^ UVA (320–395 nm) and 1.5 W/cm^2^ UVB (280–320 nm) at a path length of 3 cm using a BlueWave 75 UV Curing Spot Lamp. The area of irradiation was approximately 1 cm^2^. Following this, the samples were left for 24 h in the dark. A cell count distinguishing between viable and non-viable cells was then undertaken using trypan blue as detailed below by means of an Olympus BX50 microscope (50× objective) and a Neubauer haemocytometer as described by Wu et al. [26]. Briefly, 5 µL trypan blue stained samples (1:1 ratio) of control (unirradiated cells) and UV irradiated samples were loaded into the haemocytometer. Cells were observed for morphological changes by counting live and dead cells in 16 small squares within each of the 4 large squares and averaging the live and dead cells before comparing the total live to dead ratio to calculate the percentage viability, with each experiment repeated in triplicate. Disruption efficiency was then calculated for each irradiation duration (number of viable cells as a percentage of total cell count).

Trypan blue vitality stain was used to stain *C. reinhardtii* cells but could not be used with *D. salina* as the stain had poor miscibility in the saline media. However distinct morphological changes in *D. salina* following irradiation meant determining cell viability was possible without a stain. Additionally, Lugol’s solution was applied to *D. salina* to inhibit their motility during counting [27].

### 3.3. Lipid Extraction

As described above, samples of *C. reinhardtii* (4 samples) and *M. inermum* (8 samples) were irradiated with UV. An additional control (2 samples of *M. inermum* where only a solvent extraction was performed with no method of cellular disruption) and bead beaten samples (3 samples of *C. reinhardtii*, 2 samples of *M. inermum*) were prepared. Samples were then centrifuged (8500 rpm) and pellets retained in Eppendorfs with a 1.2 mL methanol:chloroform mixture (1:2). Supernatants were also collected to determine free lipid content.

UV exposure and control samples were kept on ice and bead beating samples were bead beaten using a cell disruptor (Genie, VWR, U.K.) for 15 cycles (1 min disruption followed by 1 min in ice, or 2 min cycles in the case of *M. inermum)* [28]. 400 µL chloroform and 400 µL ultrapure water were added to each sample before centrifuging (8500 rpm). Bottom organic phases were retained in Eppendorfs. Samples were then evaporated to dryness with nitrogen gas and reconstituted with 250 µL of methanol:chloroform mixture (1:1).

### 3.4. Transesterification

Following this, samples were transferred to glass vials and 100 µL 10% BF_3_/methanol added before incubation at 80 °C for 90 min (14 samples of *C. reinhardtii* and 24 samples of *M. inermum,* inclusive of collected supernatant samples). Samples were cooled for 10 min before addition of 300 µL ultrapure water and 600 µL hexane, then vortexed for one min. The aqueous top layers were discarded before evaporating the remaining organic phase to dryness under inert nitrogen gas using six port mini-vap evaporator (Sigma-Aldrich, Dorset, UK) [29,30]. Samples were reconstituted in 100 µL hexane and submitted for gas chromatography flame ionisation detection (GC-FID) (Thermo Finnigan TRACE 1300 GC-FID System, Thermo Scientific, Hertfordshire, UK) coupled with a TRACE™ TR-FAME GC column (25 m × 0.32 mm ID × 0.25 µm film, Thermo Scientific, Hertfordshire, UK). The experiment was repeated in duplicate.

## 4. Conclusions

Compared to a traditional cell disruption method such as bead beating, UV disruption was more efficient. Approximately 225 s of irradiation (equivalent to 335.7 J/cm^2^ UVB) resulted in 90% cell lysis of a 3 mL aliquot of *C. reinhardtii*, translating to an energy consumption of 5.6 MJ/L algae (UVB intensity at 1.5 W/cm^2^). Furthermore, GC-FID data indicated UVB radiation was more effective than bead beating for FAME yield, bead beating yielding 45.3 mg/L culture and UV irradiation yielding 79.9 mg/L culture. UV irradiation also achieved a higher FAME yield than bead beating for the thick covalently bonded cell walled species *M. inermum*, achieving a FAME yield following transesterification of extracted lipid of 226 mg/L culture compared to 208 mg/L culture for bead beating. This indicates that UV irradiation is a viable method for cell disruption to enhance lipid extraction and should be further pursued. This technology is especially promising due to its scalability because UV sterilisation for water treatment is well-established commercially.
